# Deprivation of Muscleblind-Like Proteins Causes Deficits in Cortical Neuron Distribution and Morphological Changes in Dendritic Spines and Postsynaptic Densities

**DOI:** 10.3389/fnana.2019.00075

**Published:** 2019-07-30

**Authors:** Kuang-Yung Lee, Ho-Ching Chang, Carol Seah, Li-Jen Lee

**Affiliations:** ^1^Department of Neurology, Chang Gung Memorial Hospital, Keelung, Taiwan; ^2^College of Medicine, Chang Gung University, Taoyuan, Taiwan; ^3^Graduate Institute of Anatomy and Cell Biology, College of Medicine, National Taiwan University, Taipei, Taiwan; ^4^Institute of Brain and Mind Sciences, College of Medicine, National Taiwan University, Taipei, Taiwan; ^5^Neurobiology and Cognitive Science Center, National Taiwan University, Taipei, Taiwan

**Keywords:** muscleblind-like knockouts, myotonic dystrophy, cortical neurons, interneurons, dendrites, postsynaptic densities

## Abstract

Myotonic dystrophy (Dystrophia Myotonica; DM) is the most common adult-onset muscular dystrophy and its brain symptoms seriously affect patients’ quality of life. It is caused by extended (CTG)_n_ expansions at 3′-UTR of *DMPK* gene (DM type 1, DM1) or (CCTG)_n_ repeats in the intron 1 of *CNBP* gene (DM type 2, DM2) and the sequestration of Muscleblind-like (MBNL) family proteins by transcribed (CUG)_n_ RNA hairpin is the main pathogenic mechanism for DM. The MBNL proteins are splicing factors regulating posttranscriptional RNA during development. Previously, *Mbnl* knockout (KO) mouse lines showed molecular and phenotypic evidence that recapitulate DM brains, however, detailed morphological study has not yet been accomplished. In our studies, control (*Mbnl1*^+/+^; *Mbnl2*^cond/cond^; *Nestin-Cre*^−/−^), *Mbnl2* conditional KO (2KO, *Mbnl1*^+/+^; *Mbnl2*^cond/cond^; *Nestin-Cre*^+/−^) and *Mbnl1/2* double KO (DKO, *Mbnl1*^ΔE3/ΔE3^; *Mbnl2*^cond/cond^; *Nestin-Cre*^+/−^) mice were generated by crossing three individual lines. Immunohistochemistry for evaluating density and distribution of cortical neurons; Golgi staining for depicting the dendrites/dendritic spines; and electron microscopy for analyzing postsynaptic ultrastructure were performed. We found distributional defects in cortical neurons, reduction in dendritic complexity, immature dendritic spines and alterations of postsynaptic densities (PSDs) in the mutants. In conclusion, loss of function of Mbnl1/2 caused fundamental defects affecting neuronal distribution, dendritic morphology and postsynaptic architectures that are reminiscent of predominantly immature and fetal phenotypes in DM patients.

## Introduction

Myotonic dystrophy [Dystrophia Myotonica (DM); Steinert’s disease] is the most common muscular dystrophy in adults. The prevalence rates are high in Europe and countries with European descendants (Thornton, 2014; Wenninger et al., 2018). In addition to myotonia, a delayed relaxation after forceful muscle contraction, muscle weakness/atrophy and features involving brain, heart, eyes and other organs usually accompany and worsen the course of disease. Although trials applying a variety of strategies are underway, currently no effective therapy is available (Ashizawa and Sarkar, 2011; Udd and Krahe, 2012; Thornton, 2014).

There are two types of DM: type 1 (DM1) and type 2 (DM2). Both DM1 and DM2 are microsatellite disorders that carry extended short tandem repeats within non-coding regions of different genes (Fu et al., 1992; Liquori et al., 2001). The mutations do not cause loss of function of the genes where they are located. Instead, extended (CTG)_n_ repeats in the 3′-UTR of *DMPK* gene (DM1) or (CCTG)_n_ repeats in the intron 1 of *CNBP* gene (DM2) generate pathological effects through RNA toxicity, such as stabilizing CELF1 protein and generation of repeat-associated non-ATG (RAN) proteins (Ranum and Day, 2004; Thornton, 2014; Gourdon and Meola, 2017). One of the main mechanisms is Muscleblind-like (MBNL) loss of function- When the *DMPK* gene is transcribed, the extended RNAs retain in the nuclei and formed “RNA foci,” colocalized and sequestered MBNL proteins. MBNLs are RNA-binding proteins (RBPs) that normally regulate splicing of their downstream targets during developmental transition. Once one of the targets *Clcn1* is mis-spliced, myotonia occurs due to loss of CLCN1 protein production (Miller et al., 2000; Mankodi et al., 2003; Pascual et al., 2006; Wheeler et al., 2007). It has been shown that RNA foci also exist in neuronal populations, and mis-spliced targets (e.g., GRIN1, MAPT and APP) were found in DM brains (Jiang et al., 2004). In fact, the consequence of MBNL loss of function may alter several key features of RNA processing, including polyadenylation and transportation. Therefore, it involves a very intricate underlying mechanism that is in need of further investigation (Gourdon and Meola, 2017).

Adult DM patients develop various neuropsychological symptoms including excessive daytime sleepiness, cognitive decline, depression, apathy and avoidant personality (Thornton, 2014; Minier et al., 2018). Similar to DM1, the CNS symptoms in DM2 are common but less severe. In congenital DM (CDM) with several hundreds to more than a thousand of (CTG)_n_ repeats, intellectual disability is as frequent as respiratory distress that patients may face. Autism spectrum disorder (ASD), attention deficit hyperactivity disorder (ADHD) and social difficulties are accompanied in one-third of the patients (Douniol et al., 2012; Gourdon and Meola, 2017). Brain MRI studies showed diffuse brain atrophy, predominant white matter changes in DM1 brains involving mainly frontal and temporal lobes and milder in DM2 patients (Minnerop et al., 2011, 2018; Wozniak et al., 2014). Neuropathological approaches revealed cell loss, presence of neurofibrillary tangles with tauopathy as the major findings (Caillet-Boudin et al., 2014).

Initial *in vitro* study on stable transfectans of PC12 cell line expressing extended (CTG)_90_ has shown inhibition of differentiation but not short repeats (CTG)_5_ and (CTG)_60_, suggesting the pathological (CTG)_n_ expansion is sufficient to affect terminal differentiation in neuronal cells (Quintero-Mora et al., 2002). In terms of *in vivo* models, several mouse models have been created for investigating DM mechanisms (Braz et al., 2018), but only limited transgenic lines showed brain phenotypes: one carrying DM human mutation (DM300-328 and DMSXL lines thereafter; Hernández-Hernández et al., 2013); the other is a Tamoxifen-inducible EpA960/CaMKII-Cre line which expresses 960 copies of interrupted (CTG) repeats within human *DMPK* 3′-UTR (Wang et al., 2017). Our group proved the “MBNL loss of function” hypothesis by creating knockout (KO) mouse lines of three MBNL family members (Mbnl1, Mbnl2 and Mbnl3): While *Mbnl1* KO mice (*Mbnl1*^ΔE3/ΔE3^) recapitulate myotonia and typical DM muscle pathology (Kanadia et al., 2003), *Mbnl2* constitutive KO mice (*Mbnl2* KO, *Mbnl2*^ΔE2/ΔE2^) exhibit REM sleep misregulation and cognitive dysfunction (Charizanis et al., 2012). The deprivation of Mbnl1/2 and all three members in the skeletal muscle recapitulated symptoms of muscle atrophy and respiratory distress, the end-stage DM1 or CDM features (Lee et al., 2013; Thomas et al., 2017). Based on these results, MBNL2 is considered the most critical player among the three for DM brain pathology. To investigate if loss of Mbnl1/2 may cause deleterious effects to the brain and to overcome the embryonic lethality of constitutive KO of Mbnl1/2, we chose *Nestin-Cre* mouse, a transgenic line which specifically expresses Cre recombinase in neuronal and glial cell precursors, to conditionally KO Mbnl2 specifically in the nervous system. We found combined loss of Mbnl1 and Mbnl2 in *Nestin-Cre* double KO (DKO, *Mbnl1*^ΔE3/ΔE3^; *Mbnl2*^cond/cond^; *Nestin-Cre*^+/–^) mice showed pronounced missplicing in the hippocampi similar to that of DM patients (e.g., Tau exon 2/3 and exon 10), indicating Mbnl1 may also play a compensatory role in DM brains. Since many of the downstream targets regulated by Mbnl2 were associated with the function of brain wiring (e.g., NMDAR1 or GRIN1, MAPT and DCLK1; Shin et al., 2013; Jadhav et al., 2015; Perez-Rando et al., 2017), we wondered if structural changes of cortical neurons may also be present in our *Mbnl* KO lines.

## Materials and Methods

### *Mbnl* KO Mouse Lines

Original articles describing the generation of *Mbnl1* KO (*Mbnl1*^ΔE3/ΔE3^), *Mbnl2* neuron-specific conditional KO (*Mbnl2* CKO or 2KO; *Mbnl2*^cond/cond^; *Nestin-Cre*^+/−^) and *Nestin-Cre* DKO (*Mbnl1/2* DKO or DKO; *Mbnl1*^ΔE3/ΔE3^; *Mbnl2*^cond/cond^; Nestin-Cre^+/−^) were published (Kanadia et al., 2003; Charizanis et al., 2012; Goodwin et al., 2015). Following the protocols we developed in the University of Florida, mice were generated by crossing two single KO lines in the animal center of Chang Gung Memorial Hospital, Keelung, Taiwan, one of the AAALAC accredited institutes that is conducting serious medical research. Due to the breeding difficulties and reduced body weight of the DKO mice, we had to delay weaning and the tail clipping/genotyping process until 5–6 weeks after their birth. In addition, the lifespans of the DKO mice were shortened and they could rarely survive longer than 5 months. Therefore, 2–4-month-old mutant mice were collected in this study. Age-matched mice of *Mbnl1*^+/+^; *Mbnl2*^cond/cond^; *Nestin-Cre*^−/−^ were used as controls. The number of mice used and experimental procedures were reviewed and approved by the IACUC committee (IACUC No. 2015121903).

### Preparation of Brain Tissues

Mice were anesthetized with sodium pentobarbital (50 mg/kg i.p.) and perfused with phosphate-buffered saline (PBS), followed by 4% paraformaldehyde (for immunohistochemistry and Golgi staining) or 2% glutaraldehyde and 2% paraformaldehyde (for electron microscopy). Brains were collected and half brains were cut into 40 μm (for immunohistochemistry) or 100 μm (for electron microscopy) using a vibrating microtome (VT1000S, Leica Biosystems, Wetzlar, Germany). Paraformaldehyde-fixed remaining brain halves were used for Golgi staining.

### Immunohistochemistry

Coronal sections 40 μm-thick containing the sensorimotor cortex were treated with 0.3% H_2_O_2_ in PBS to block the endogenous peroxidase activity, followed by treating with blocking solution to reduce the non-specific bindings. The sections were then incubated with primary antibodies against NeuN (1:500; Merck Millipore, Darmstadt, Germany), Foxp1 (1:1,000; Abcam, Cambridge, UK), Cux1 (1:1,000; Santa Cruz Biotechnology, Dallas, TX, USA), GAD65/67 (1:2,000; Sigma, St. Louis, MO, USA), Parvalbumin (Pvalb or PV, 1:3,000; Sigma) and Calretinin (Calb2 or CR, 1:3,000; Merck Millipore) at room temperature overnight. Next, sections were incubated with biotinylated secondary antibodies against mouse or rabbit IgG (1:500; The Jackson ImmunoResearch Laboratories, West Grove, PA, USA) for 2 h at room temperature followed by Vectastain [avidin-biotin complex (ABC) kit, Vector Laboratories, Burlingame, CA, USA] incubation for 1 h. Last, sections were processed with 2 mg/ml of 3,3′-Diaminobenzidine (DAB) with 0.01% H_2_O_2_ in PBS and mounted with a glycerol-based aqueous mounting medium.

### Measurement of Cortical Neurons

To measure the density of immunochemistry-labeled neurons in the cortex, we counted the immunopositive cells within each counting square of 100 μm × 100 μm in the upper, middle and lower regions of the sensorimotor cortex. To estimate the relative distribution of cortical neurons, the thickness of the cortex was equally subdivided into 10 counting bins, starting from the pia surface (top) to the border of white matter (bottom). The width of the counting bins was 50 μm in counting NeuN-, Foxp1- and Cux1-positive neurons and 100 μm in counting GAD65/67-, PV and CR-positive neurons, respectively. In this counting system, bin 1 corresponds to cortical layer I, bins 2–3 to layers II/III, bin 4 to layer IV, bins 5–7 to layer V and bins 8–10 to layer VI (Yu et al., 2019). The number of cells was counted and the percentages of each counting bins were calculated.

### Golgi Staining

Brain samples were impregnated and processed as previously described (Li et al., 2014). In brief, paraformaldehyde-fixed brain halves were kept in the impregnation solution of FD Rapid GolgiStain kit (NeuroTechnologies, Ellicott City, MD, USA) for 3 weeks. After being washed with ddH_2_O, the 100 μm sections were acquired using a vibratome (Leica), followed by incubation with developer and fixer solutions and finally mounted on gelatin-coated slides. The images of Golgi-stained layers II/III pyramidal neurons in the sensorimotor cortex were captured by the StereoInvestigator system (MicroBrightField Bioscience, Williston, VT, USA) using a light microscope (Olympus, Tokyo, Japan). The soma and dendritic structures of collected neurons were reconstructed from stacks of images using the Neurolucida system (MicroBrightField Bioscience). Size-related parameters (dendritic length) and topological parameters (numbers of branching nodes, terminal endings and dendritic segments) were subsequently analyzed. The complexity of dendrites was determined by counting the number of dendritic segments of different orders, intersections between dendritic branches and the concentric rings method (Sholl analysis; Wang Y. C. et al., 2012). Any protrusion was defined as spine and the spine density was calculated and shown as numbers per micrometer of dendritic length. Dendritic spines were further classified by their morphology based on the published criteria: mushroom (spine head greater than 0.6 μm in diameter), stubby (short without a head), and thin (long with a head less than 0.6 μm in diameter) and filopodia (long without a head; Sorra and Harris, 2000). The length and width of individual spines were also measured.

### Transmission Electron Microscopy

The sections of 100 μm thickness containing the sensorimotor cortex were collected from Bregma 0.98 mm to 0.14 mm and post-fixed with 1% aqueous osmium tetroxide for 1 h. The upper portions comprising cortical layers II/III were isolated for further processes. The samples were dehydrated in graded ethanol, washed with propylene oxide, and embedded in epoxy resin (Polysciences Inc., Warrington, PA, USA). Semi- and ultra-thin sections were cut with a diamond knife on a Reichert-Jung Ultracut E ultramicrotome (Leica-Microsystems, Wetzlar, Germany). Ultra-thin sections were collected on copper grids (200 mesh, TAAB, Burks, UK) and stained with lead citrate and uranyl acetate. Digital photographs at a magnification of 10,000× were obtained by a transmission electron microscope (Hitachi H-7100, Tokyo, Japan) at 100 kV. The thickness and width of the postsynaptic densities (PSDs) were measured using ImageJ software (NIH, Bethesda, MD, USA). The glutamatergic synapses were selected and the grayscale values across the synapses were measured. The PSD thickness was defined as the distance between the grayscale values of postsynaptic local maximum and the first local minimum. The PSD width was defined as the length of electron dense band along the postsynatic membrane (Jiang-Xie et al., 2014).

### Statistical Analysis

Statistical tests were performed using SPSS software (IBM, Armonk, NY, USA). All data were analyzed by one-way ANOVA followed by Scheffe’s *post hoc* analyses and *p* < 0.05 was defined as the statistical significance. All data were expressed as mean ± SEM and the *n* values of each experiment were noted in the Figure legends.

## Results

### Mbnl Loss of Function Caused Abnormal Distribution of Cortical Neurons

Neuroimaging studies have revealed structural abnormalities in the gray and white matter of the sensorimotor cortex associated with the CNS malfunctions in DM1 patients (Minnerop et al., 2018).

**Figure 1:**
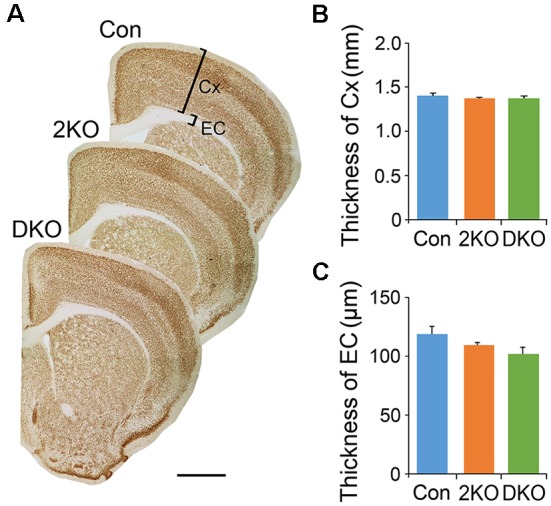
Thicknesses of gray matter and white matter in *Mbnl* mutant mice. The gray matter in the sensorimotor cortex (Cx) and the white matter underneath, the external capsule (EC), were measured in sections collected from adult male control (Con, *n* = 7), *Mbnl2* conditional knockout (CKO; 2KO, *n* = 7) and *Mbnl1/2* double KO (DKO, *n* = 6) mice **(A)**. Significant differences were not reached in the thicknesses of both Cx **(B)** and EC **(C)** among mice of three different genotypes. Bar = 1 mm. Results are mean ± SEM.

We first examined the features in the cortex of 2KO and DKO mice, the brain-specific mouse models for DM. We measured the thicknesses of the sensorimotor cortex (gray matter) and the external capsule (EC; white matter) underneath in these mice (Figure 1A). One-way ANOVA showed no significant difference in the thickness of cortex (*F*_(2,12)_ = 0.471, *p* = 0.63; Figure 1B) and the EC (*F*_(2,17)_ = 2.492, *p* = 0.112; Figure 1C) among the control, 2KO and DKO mice.

**Figure 2:**
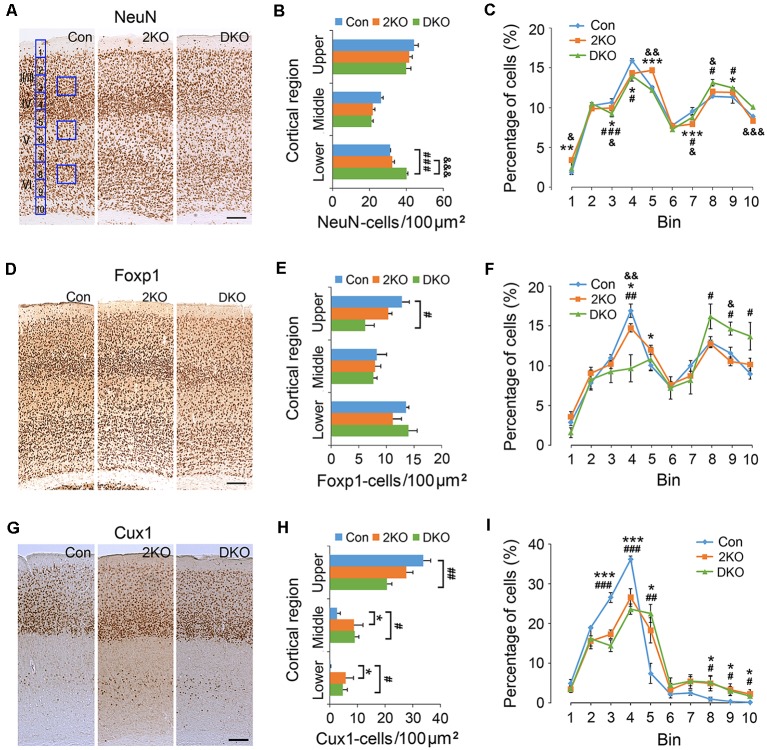
Density and distribution of cortical neurons in *Mbnl* mutant mice. Coronal sections containing the sensorimotor cortex of control (Con, *n* = 4), *Mbnl2* CKO (2KO, *n* = 4) and *Mbnl1/2* DKO mice (DKO, *n* = 3) mice were immunostained with NeuN **(A)**, Foxp1 **(D)** and Cux1 **(G)**. To determine the neuronal densities, counting squares of 100 μm × 100 μm were assigned to the upper, middle and lower cortical regions, and the numbers of NeuN-, Foxp1- and Cux1-positive neurons within each counting square were quantified **(B,E,H)**. Next, to measure the distribution of immunolabeled cortical neurons, the thickness of the cortex was equally divided into 10 counting bins with the width of 50 μm, and the percentages of immunolabeled neurons were quantified **(C,F,I)**. Bar = 100 μm. Results are mean ± SEM. Asterisk (*), hash (^#^) or and (^&^) indicate significant differences between Con and 2KO mice, Con and DKO, 2KO and DKO, respectively (*,^#^,^&^*p* < 0.05; **,^##^,^&&^*p* < 0.01; ***,^###^,^&&&^*p* < 0.001; two-tailed *t*-test).

Next, we examined the density and distribution of cortical neurons in the sensorimotor cortex of these mice. The density of cortical neurons labeled with a pan-neuronal marker, NeuN, was measured in 100 μm × 100 μm counting frames in the upper (layers II–IV), middle (layer V) and lower (layer VI) cortical regions (Figures 2A,B). We found no significant difference in the densities in the upper (*F*_(2,8)_ = 2.914, *p* = 0.112) and middle (*F*_(2,8)_ = 3.522, *p* = 0.08) regions among three genotypes. In the lower cortical region (*F*_(2,8)_ = 33.225, *p* < 0.001); the density of NeuN-positive neurons largely increased in DKO mice, compared with control (*p* < 0.001) and the 2KO (*p* < 0.001) mice (Figure 2B). For a detailed analysis of cell distribution, we checked NeuN-positive neurons in the cortex by dividing the thickness of cortex into 10 counting bins and measuring the number of cells within each bin (Figure 2A). Compared with control mice, the percentages in bin 3 and 4 (corresponding to layers II/III and IV) decreased (2KO *p* < 0.05, DKO *p* < 0.001) but slightly increased in bin 9 (layer VI) in both mutants (*p* < 0.05; Figure 2C).

To confirm these findings, we evaluated the expression of Foxp1, a member of the FOX transcription factors and a marker for forebrain pyramidal neurons (Figures 2D–F; Ferland et al., 2003). In the upper cortical region (*F*_(2,6)_ = 6.713, *p* < 0.05); the density of Foxp1-positive neurons largely (~50%) decreased in the DKO mice compared with controls (*p* < 0.05; Figure 2E). In the middle (*F*_(2,6)_ = 1.355, *p* = 0.327) and lower (*F*_(2,6)_ = 0.6, *p* = 0.943) cortical regions, the difference among groups was not significant. Distribution analysis revealed a distinct decrease of the Foxp1-positive neurons in bin 4 in the DKOs (*p* < 0.01), and a subtle reduction in the 2KO mice (*p* < 0.05). In addition, the percentages in bins 8–10 (layer VI) increased in the DKO mice (*p* < 0.05; Figure 2F). We also double-checked our findings by choosing Cux1, a homeobox transcription factor normally expressed in upper cortical neurons (Cubelos et al., 2010) for the immunostaining studies (Figures 2G–I). Compared with control mice, the density of Cux1-positive cells again decreased in the upper and increased in the middle and lower regions in the DKOs (*p* < 0.01, *p* < 0.05, *p* < 0.05, respectively; Figure 2H). In terms of distribution, we also found an abnormal reduction in bins 3 and 4 (layers II/III and IV; 2KO, *p* < 0.001 and DKO, *p* < 0.001) but an increase in bin 5 (layer V; 2KO, *p* < 0.05 and DKO, *p* < 0.01) and in bins 8–10 (layer VI) in both mutant mice (*p* < 0.05; Figure 2I). These results indicated a failure of Cux1-positive cortical neurons to reach their final destinations in the mutants. Taken together, our results suggested an altered distribution of glutamatergic cortical neurons in both mutants, more prominent in the DKO mice.

### Density and Distribution of Inhibitory Interneurons in the Cortex

**Figure 3:**
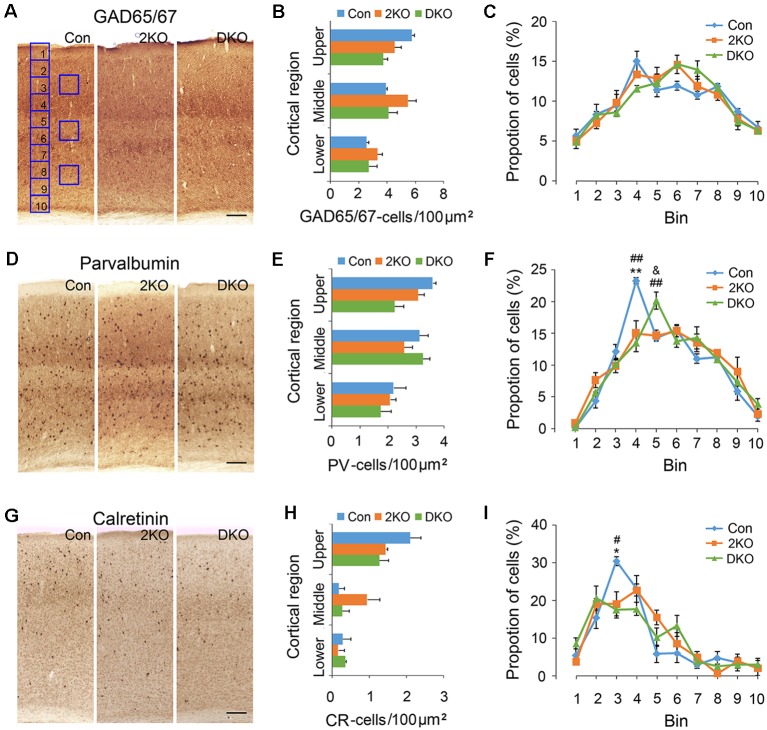
Densities and distribution of GABAergic interneurons in *Mbnl* mutant mice. Coronal sections containing the sensorimotor cortex of male adult control (Con, *n* = 4), *Mbnl2* CKO (2KO, *n* = 4) and *Mbnl1/2* DKO mice (DKO, *n* = 3) mice were immunostained with GAD65/67 **(A)**, Parvalbumin, PV **(D)** and Calretinin, CR **(G)**. To determine the neuronal densities, counting squares of 100 μm × 100 μm were assigned to the upper, middle and lower cortical regions, and the numbers of GAD65/67-, PV- and CR-positive neurons within each counting square were quantified **(B,E,H)**. Next, to measure the distribution of immunolabeled cortical neurons, the thickness of the cortex was equally divided into 10 counting bins with the width of 100 μm and the percentages of immunolabeled neurons were quantified **(C,F,I)**. Bar = 100 μm. Results are mean ± SEM. Asterisk (*), hash (^#^) or and (^&^) indicate significant differences between Con and 2KO mice, Con and DKO, 2KO and DKO, respectively (*,^#^,^&^, *p* < 0.05; **,^##^*p* < 0.01).

GABAergic inhibitory interneurons in the cortex play important roles in the processing of neuronal information. Immunostaining of GAD65/67 was performed for labeling GABAergic interneurons (Figures 3A–C), while antibodies against PV (Figures 3D–F) and CR (Figures 3G–I) were used to label two separated subgroups of interneurons (Salaj et al., 2015). We found the density of GAD65/67-positive cells in the upper cortical region was reduced in DKO mice, although the results did not achieve statistical significance (*p* = 0.057; Figure 3B). On the other hand, the distribution of GAD65/67-positive neurons evaluated by 10 counting bins did not significantly change in either 2KO or DKO mice (Figure 3C). Alternatively, using different markers, we found a reduced density of PV-positive interneurons in DKO mice, but this still did not reach significance (*p* = 0.062; Figure 3E). However, the distribution analysis revealed a reduction of the percentage of PV-positive cortical interneurons in bin 4 in both 2KO and DKO mice compared to controls (both *p* < 0.01; Figure 3F). In addition, although only a trend of reduction was observed in the mutants during the density analysis for CR-positive interneurons (Figure 3H); the percentage of CR-positive cortical interneurons in bin 3 significantly decreased in 2KO and DKO mice compared to the control mice during distribution analysis (both *p* < 0.05; Figure 3I). Taken together, these results may suggest a milder distributional defect also exists in GABAergic cortical interneurons of both KO mice.

### Reduced Dendritic Complexity in the *Mbnl* KO Mouse Models

**Figure 4:**
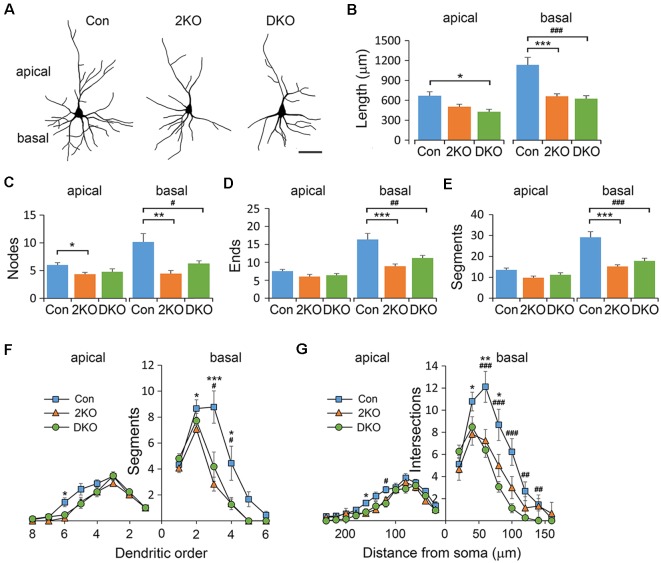
Dendritic architectures of layer II/III pyramidal neurons in *Mbnl* mutant mice. Golgi-impregnated layers II/III pyramidal neurons were collected from male adult control (Con), *Mbnl2* CKO (2KO) and *Mbnl1/2* DKO (DKO) mice and reconstructed **(A)**. The dendritic length **(B)**, number of bifurcation nodes **(C)**, number of terminal ends **(D)** and number of dendritic segments **(E)** was measured in both apical and basal dendrites. The complexity of the branching pattern in apical and basal dendrites was examined by counting the number of segments in different orders **(F)**, and using Sholl’s concentric ring method **(G)**. Bar = 20 μm. Data is mean ± SEM. For apical dendrites, *n* = 21 neurons from four Con mice; 9 neurons from three 2KO mice; 13 neurons from three DKO mice. For basal dendrites, *n* = 11 neurons from three Cons, 12 neurons from three 2KOs; 15 neurons from three DKOs. Asterisk (*) or hash (^#^) indicate significant differences between Con and 2KO or Con and DKO, respectively (*,^#^*p* < 0.05; **,^##^*p* < 0.01; ***,^###^*p* < 0.001).

To evaluate the cytoarchitecture of glutamatergic pyramidal neurons, we examined the apical (emerged upward from the apex) and basal (emerged downward from base) dendrites in layers II/III pyramidal neurons, collected from the sensorimotor cortex (Figure 4A). Significant differences in dendritic length were noted in both apical (*F*_(2,40)_ = 5.378, *p* < 0.01) and basal (*F*_(2,33)_ = 17.944, *p* < 0.001) dendrites. Compared with that of the control group, the length of apical dendrites was reduced in the DKO mice (*p* < 0.01), while the length of basal dendrites was largely reduced in both mutants (*p* < 0.001; Figure 4B). Furthermore, the number of nodes, ends and segments decreased in the basal, but rather mildly, in apical dendrites of both mutants (Figures 4C–E). We then measured the complexity of neurons using the aforementioned methods. We found that in both mutants, the dendritic complexity was significantly reduced in the basal dendrites but much less prominent in the apical dendrites (Figures 4F,G). For most of the categories in this analysis, the effects of single KO Mbnl2 and combined KO Mbnl1/2 were very similar, especially in the basal dendrites. These results suggested Mbnl2 loss of function alone was sufficient to disrupt the dendritic morphogenesis in cortical neurons.

### Immature Dendritic Spines Increased in the Absence of Mbnl 1/2

**Figure 5:**
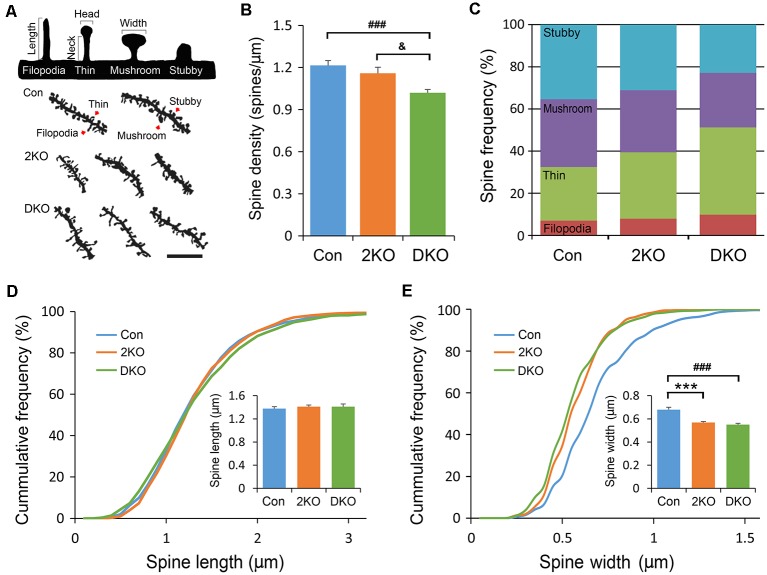
Structural analysis of dendritic spines. Golgi-impregnated dendritic spines in the layers II/III pyramidal neurons in the sensorimotor cortex were imaged and measured. Dendritic segments were obtained from male adult control (Con) and *Mbnl* mutant mice (2KO and DKO). Dendritic spines were classified into stubby, mushroom, thin and filopodia as illustrated with definitions of head/neck and length/width in upper panel. Representative dendrites with different types of dendritic spines (indicated by arrows) are shown in the lower panel **(A)**. By counting the spine numbers, the density of dendritic spines decreased significantly in the DKO group **(B)**. The frequencies of spines with different configurations were calculated. **(C)** While the lengths of dendritic spines were similar among three groups** (D)**, the widths of dendritic spines were significantly reduced in 2KO and DKO groups **(E)**. Bar = 5 μm. Data is mean ± SEM. In Con group, 1,145 spines were collected from 25 neurons in three mice; in the 2KO group, 947 spines were obtained from 22 neurons in three mice; in the DKO group, 1,652 spines were collected from 27 neurons in three mice. Asterisk (*), hash (^#^) or and (^&^) indicate significant differences between Con and 2KO mice, Con and DKO, 2KO and DKO, respectively (^&^*p* < 0.01; ***,^###^*p* < 0.001).

Dendritic spines are tiny protrusions from dendritic shafts that are critical for receiving excitatory inputs from glutamatergic neurons. Their morphology may change during development or in response to neuronal activity and are directly linked to synaptic function. Neuroscientists developed methods to classify the dendritic spines into stubby, mushroom, thin and filopodia. The different morphology may reflect their maturity: the stubby and mushroom-like spines are mature and well-developed, whereas the thin and filopodia spines are relatively immature and hypogenetic. We then characterized the spine densities and analyzed their morphology under high power magnification with a light microscope (Figure 5A). Significant difference in spine density was noted among three groups (*F*_(2,81)_ = 10.612, *p* < 0.001); but only significantly decreased in neurons of the DKO mice, compared with those in control (*p* < 0.001) and 2KO (*p* < 0.05) mice (Figure 5B). We further compared the frequencies of different spine types among three genotypes. Significant differences were noted in stubby (*F*_(2,72)_ = 14.415, *p* < 0.001) and thin (*F*_(2,72)_ = 18.82, *p* < 0.001) spines. The frequency of stubby type (mature) spines was significantly reduced in the DKO group (22.9%) compared with control (35.3%, *p* < 0.001) and 2KO (31.2%, *p* < 0.01) groups, respectively. On the other hand, the ratio of thin type (immature) spines was largely increased in the DKO group (41.3%) compared with control (25.44%, *p* < 0.001) and 2KO (31.5%, *p* < 0.01) groups (Figure 5C). These results indicated a defect in the maturation of dendritic spines in the DKO mice. We also measured the length and width of each dendritic spine. The spine length was comparable among three groups (*F*_(2,71)_ = 0.254 *p* = 0.776; Figure 5D); while the width of dendritic spines decreased in both 2KO and DKO groups compared with controls (*p* < 0.001 for both mutants; Figure 5E). These results indicate a shared feature of defective spines and compromised synaptogenesis in these mutants, more severe in the DKO mice.

### Alterations of the Postsynaptic Density (PSD) Morphology in the DKO Mice

**Figure 6:**
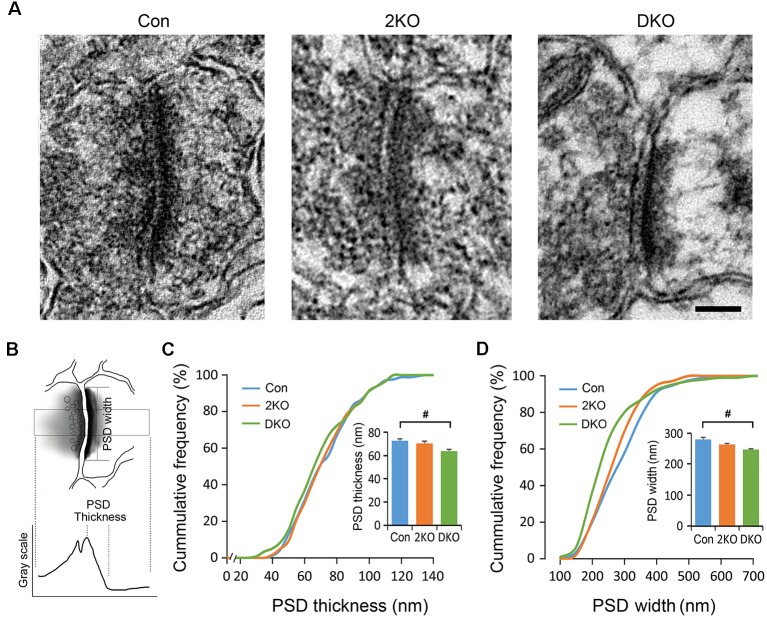
Electron microscopic analysis of synaptic ultrastructures in *Mbnl* KO mouse models. Electron micrographs of the postsynaptic densities (PSDs) in the sensorimotor cortex were collected from male adult control (Con, *n* = 4), *Mbnl2* CKO (2KO, *n* = 4) and *Mbnl1/2* DKO (DKO, *n* = 3) mice **(A)**. By our definition, the PSD thickness is the distance between local maximum and minimum of grayscale values across a synapse; and the PSD width is the electron dense band along the postsynaptic membrane **(B)**. The PSD thickness depicted in cumulative frequency revealed reduced thickness in the DKO mice **(C)**. The PSD width depicted in cumulative frequency showed significant reduction with the curve shifted to the left in the DKO group **(D)**. Bar = 100 nm. Data are mean ± SEM. Hash (^#^) or and (^&^) indicate significant differences between Con and DKO, 2KO and DKO, respectively (^#^*p* < 0.05).

The PSDs consist of various postsynaptic receptors and scaffold proteins and the features of PSDs are highly linked to synaptic function. We therefore decided to evaluate the ultrastructure of the PSDs of excitatory synapses in the sensorimotor cortex using electron microscopy (Figure 6). The PSDs of excitatory synapses in the sensorimotor cortex were collected from control, 2KO and DKO mice (Figure 6A). The thickness of PSD was defined as the distance between the local maximum and minimum of postsynaptic grayscale values across a glutamatergic synapse, whereas the PSD width was the length of the high electron-dense band along the postsynaptic membrane (Figure 6B). Although the changes in the 2KO mice were subtle and did not reach significant differences, as expected, we found a significant reduction of PSD thickness and widths in the DKO mice compared to control mice (*p* < 0.05; Figures 6C,D). These results indicate loss of Mbnl would potentially alter the synaptic transmission, particularly in the DKOs.

## Discussion

MBNL family proteins are RBPs critical for post-transcriptional regulations (Batra et al., 2015; Taylor et al., 2018). Aside from the RNA toxicity derived from microsatellite repeat expansions including RAN translation and secondary CELF1 upregulation (Wang et al., 2007, 2009; Zu et al., 2011, 2017), MBNL loss of function is essential for causing DM phenotypes(Thomas et al., 2017; Chen et al., 2018; Ramon-Duaso et al., 2019). The 2KO and DKO models have shown their potential and suitability for the investigations of DM brain pathogenesis due to extensive and distinct splicing alterations similar to DM patients (Charizanis et al., 2012; Goodwin et al., 2015). However, detailed morphological evaluation has been lacking. Therefore, this project was aiming to look for structural evidence at the cellular level in these mouse models.

The macroscopic studies on DM1 adult patients were generally normal, without characterizing significant brain atrophy. However, in the longitudinal studies of air encephalography, progressive atrophy did occur in adult DM1 patients (Refsum et al., 1967). On the other hand, the severity of brain atrophy was higher in CDM patients and the observations were further confirmed by most series of CT or MRI image studies showing ventricular dilatation and marked brain atrophy (Regev et al., 1987). In our results, we were unable to see major changes in gray and white matter thickness. Although a trend of reduced EC thickness in the mutants was observed, it was unable to reach statistical significance. This may be a result of the relatively young mice selected (2–4 months, due to the reduced life expectancy of the DKOs) of our experimental animals or limited numbers of brain checked. Nevertheless, our results did not notably deviate from the previous findings in adult DM patients.

Neuronal migration is a critical step during cortical development for neurons to accomplish proper positioning and form brain circuitry. During the early embryonic stage, newborn cortical neurons starting from the ventricular zone move radially alongside with radial glial fibers to the cortical plate and form six cortical layers in an inside-out manner (Pilz et al., 2002; Marin et al., 2010). Early cortical plate neurons that settle in layer VI become the corticothalamic projection neurons while layer V pyramidal neurons are mainly corticospinal projection neurons. Layers IV, III and II neurons receive thalamic inputs and projections from other cortical regions and layers (Petreanu et al., 2009). Migration defects could cause detrimental effects and may potentially be linked to neuropsychiatric disorders such as autism (Reiner et al., 2016). Recently, neuroscientists have started to realize that RBPs may potentially regulate migration. For example Nova2, a splicing factor, has shown its function in modulating migration for the upper layer cortical neurons in mice, suggesting the importance of RBPs during this process (Yano et al., 2010; Vuong et al., 2016). Previous reports on gyral architecture in CDM patients and if migratory defects exist in DM were under debate. However, a report showing multiple cases with poor lamination of cortex and architectural arrangement of neurons was published more than half a century ago (Rosman and Kakulas, 1966; Hageman et al., 1993).

In our study, a considerable amount of cortical neurons, such as Cux1-positive upper cortical neurons, failed to reach their final destination in the mutants, suggesting a defect in cortical neuron migration. Unlike glutamatergic cortical neurons, GABAergic inhibitory interneurons derive from another proliferative region, the ganglionic eminence, in the ventral telencephalon. PV-positive interneurons, which play an important role in the gating of sensory-motor circuits (Sachidhanandam et al., 2016), are mostly derived from the medial ganglionic eminence; whereas CR-positive interneurons are largely derived from the caudal ganglionic eminence (Cauli et al., 2014). During development, inhibitory cortical interneurons first migrate dorsally and then tangentially toward the cortex (Marin, 2013). The patterns of PV- and CR-positive cells in the upper cortical regions of our KO mice suggested a relatively mild defect in the distribution of cortical interneurons as well. Our results demonstrated for the first time that loss of function of Mbnl proteins may cause abnormalities in neuronal distribution, which may be caused by migratory alteration. To further confirm if these changes were directly caused by migration deficits rather than a shift in the neuronal lineage, alteration of progenitor proliferation or a result of apoptotic cell death, BrdU pulse-chase experiments would be necessary in the future to directly answer this question.

Positioned cortical neurons initiate dendrite and axon identities postnatally for their specific input/output functions after migration. The growth and formation of dendrites is a fundamental process for proper synaptic connections, equally important as axonal maturation. Dendritic structures are constantly changing, under the influence of experience-dependent plasticity (Maravall et al., 2004). Alterations of dendritic morphology have also been reported in neuropsychiatric diseases, such as Fragile X syndrome (Irwin et al., 2001). In our morphological studies on dendrites, we chose parameters that were functionally relevant. For example, the number of branch orders (bifurcation nodes) may influence signal propagations (Brown et al., 2008). Surprisingly, the impact of lacking Mnbl proteins on apical and basal dendrites was different. We observed greater structural changes in the basal dendrites than the apical dendrites in layers II/III pyramidal neurons of both 2KO and DKO mice. Layers II/III basal dendrites receive glutamatergic excitatory inputs from the thalamus as well as layer VI neurons and the branching patterns are sensitive to sensory experience (Bose et al., 2010). Our results suggest Mbnl loss of function may alter this feedforward signals in the sensory-motor circuitry (Petreanu et al., 2009). Interestingly, the defective basal dendrites in layers II/III cortical neurons were evident and comparable between 2KO and DKO lines, indicating the predominant role of Mbnl2 in determining dendritic branching.

Apical and basal dendrites are functionally specified structures. The molecular mechanisms underlying the formation and arborization of apical and basal dendrites are just about to be revealed (Chow et al., 2009). For example, TAOK2 and EPAC2 are ASD—related genes. EPAC2 is a guanine nucleotide exchange factor (GEF) for the Ras-like small GTPase Rap, downstream of sema3A-Npn1-PlexinA4 signaling cascade, which is activated by TAOK2 (Srivastava et al., 2012; de Anda et al., 2012). Both *Epac2* knockdown and *Taok2* KO mice exhibit poor basal dendrite complexity yet relatively normal apical dendrites in layers II/III neurons (Srivastava et al., 2012; de Anda et al., 2012), similar to our findings. These phenotypes of basal dendrites may be a shared feature in disease showing autism-associated symptoms, including DM1/CDM (Angeard et al., 2018). Dendritic spines are micron-scale protrusions from dendritic shafts and the sites where most excitatory synapses are located. These dendritic spines functionally connect nerve fibers, which are fundamental for brain circuits. The structure of dendritic spines is dynamic and influenced by synaptic activity. Neurotrophic factors and extracellular matrix glycoproteins are key players that regulate their formation. They are crucial for learning and memory but may be severely damaged under pathological conditions (Arikkath, 2012; Adrian et al., 2014). During spinogenesis, the number of spines were quite few in fetal or newborn animals. The immature appendage/filopodia/filopodia-like spines emerged first and were followed by mature spines later postnatally (García-López et al., 2010). Compared to the length that is more specifically linked to calcium compartmentation and calcium-dependent learning, the width of the spine has a stronger correlation with the synaptic strength (Arellano et al., 2007). Therefore, the primary width alterations suggested the indispensable roles of Mbnl1/2 in maintaining synaptic strength.

Impaired hippocampal electrophysiological activity and neuronal circuits have been identified in the *Mbnl2* constitutive KO mice (Charizanis et al., 2012; Chen et al., 2018; Ramon-Duaso et al., 2019). Since dendritic spine morphology is correlated with synaptic function, we applied electron microscopy to evaluate PSDs that were most likely affected by impaired dendritic compositions (Arellano et al., 2007). The PSDs are composed of a variety of proteins including glutamatergic receptors and adhesion molecules, located at the tip of dendritic spines and critical for maintaining structural and physiological integrity of the synapses (Sheng and Hoogenraad, 2007). As expected, the thickness and width of PSDs were reduced in the DKO mice, which is compatible with the results found in dendritic spine analysis, suggesting defects in synaptic transmission and plasticity may occur in these mutants.

Based on previously published data, Mbnl1 and Mbnl2 are highly expressed in the neurons. The distribution of Mbnl1 and Mbnl2 are both nuclear and cytoplasmic (Jiang et al., 2004; Charizanis et al., 2012; Wang E. T. et al., 2012). The distribution of Mbnl proteins may differ in different cell types. For example, the Mbnl1 expression in the cerebellar Purkinje neurons is exclusively cytoplasmic, while in the cortical neurons, cerebellar molecular interneurons and deep cerebellar nuclei neurons, the Mbnl1 expression is both nuclear and cytoplasmic. On the other hand, Mbnl2 is highly expressed in the nucleus of neurons in the frontal cortex (Jiang et al., 2004; Daughters et al., 2009; Charizanis et al., 2012; Wang et al., 2017).

KO mouse models have demonstrated the functions of Mbnl proteins. The overall splicing changes were very mild in *Mbnl1* KO but readily detectable through RT-PCR, microarray and RNAseq experiments in *Mbnl2* KO. The behavioral evaluation of *Mbnl1* KO was limited due to its constitutive KO and marked myotonia that may affect locomotion. On the other hand, we saw spatial learning deficits as well as eletrophysiological changes in *Mbnl2* KO mice. Therefore, we thought MBNL2 is the key player in DM brains, in contrast to the relative predominant role of MBNL1 in the skeletal muscle. However, this does not mean an exclusive tissue-specific role for individual MBNL. Evidence from *Myo-Cre* DKO that KO of Mbnl1/2 in muscle shows severe muscular atrophy and *Nestin-Cre* DKO that KO of Mbnl1/2 in the nervous system shows complete Tau spliceopathy suggest compensatory roles Mbnl1 and Mbnl2 in different organs (Lee et al., 2013; Goodwin et al., 2015).

In addition to nuclear MBNL that regulate splicing, both cytoplasmic Mbnl1 and Mbnl2 may participate in RNA processing such as RNA localization and facilitate local translation. For example, cytoplasmic Mbnl1 binds to 3′-UTR of downstream gene targets and transports them to the membrane organelles, including the synapses (Adereth et al., 2005; Wang E. T. et al., 2012). Of note, the nuclear Mbnl1 splicing regulation may also be associated with 3′-UTR binding, since spliced transcripts regulated by Mbnl1 have twofold chances of 3′-UTR binding by Mbnl1, suggesting a collaborative work between nuclear and cytoplasmic Mbnl1. The potential cytoplasmic function of Mbnl1 is intriguing especially in the asymmetrical cells such as neurons, since focal structures far away from the nucleus, such as axon and synapse may be more sensitive to RNA localization. The enriched Mbnl1 binding sites in the synapse suggest its potential role in regulating synaptic function (Wang E. T. et al., 2012).

It has been known that nuclear Mbnl1 and Mbnl2 autoregulate splicing of themselves, including exon 7 (54 nt) of Mbnl1 and exon 6 (54 nt) of Mbnl2 with sequence of nuclear localizing signals. This translocation from cytoplasm into nucleus is especially important during developmental transition from fetal to adult (Lin et al., 2006). In the *Mbnl* KO mice, the splicing shifted these alternative-spliced cassettes towards exon-inclusion, therefore the nuclear portion of Mbnl1 and Mbnl2 was increased (Lee et al., 2013). Interestingly, it was reported that an alternative post-translational modification pathway may determine the translocation of Mbnl1 from cytoplasm to nucleus by deubiquitination (Wang et al., 2018). In the transgenic mice overexpressing (CTG)_960_ specifically in the brain, they found reduced cytoplasmic Mbnl1 is an early event and this cytoplasmic isoform is necessary for maintaining neurite growth. In addition, loss of function of cytoplasmic Mbnl1 showed negative impact on the synaptic transmission, prior to the nuclear Mbnl2-mediated mis-splicing of downstream targets. Therefore, the mechanism and impact of loss of Mbnl1 or Mbnl2 on the dendrite growth may be different (Wang et al., 2017, 2018). In our *in vivo* system, we chose CKO strategy in order to get DKO mice in the nervous system. Initially, we did not include *Mbnl1* KO due to its subtle brain phenotype as we reported previously (Suenaga et al., 2012). Although Mbnl1 plays a predominant role in the cytoplasm and Mbnl2 functions more significantly in the nuclei, our observation on *Nestin-Cre* DKO nevertheless revealed a phenotype that contributed by compound loss of MBNL1/2 in the nervous system. To compare and verify specific consequences of loss of Mbnl1 or Mbnl2 to the dendrite/dendritic spines, further work on morphological analysis will be worth doing by using individual *Mbnl* KO lines.

In conclusion, through the morphological studies on *Mbnl* KO models, we once again proved that Mbnl1/2 proteins play a critical role in the developing brain through affecting the distribution patterns of cortical projection neurons and inhibitory interneurons as well as dendritic complexity, spine morphology and PSD ultrastructures. The Mbnl1/2 loss of function caused the infant/immature structural phenotypes in mutant brains that were reminiscent of the fetal isoform retention in adults during splicing analysis (Charizanis et al., 2012). These deficits may alter the synaptic transmission and brain circuitry in KO mice and potentially also in DM patients. Our findings provided the first morphological evidence of compromised neurons in *Mbnl* KO brains that will be very helpful in understanding CNS pathogenesis in DM.

## Data Availability

All datasets generated for this study are included in the manuscript.

## Ethics Statement

Mice were generated in the animal center of Chang Gung Memorial Hospital, Keelung, Taiwan, an AAALAC accredited institute. To maintain the highest standard of animal welfare, the IACUC committee ensured the number of mice used was reduced and the experimental procedures were refined (IACUC No. 2015121903).

## Author Contributions

H-CC and CS conducted the experiments and analyzed the data. K-YL and L-JL designed the experiments, analyzed the data and prepared the manuscript.

## Conflict of Interest Statement

The authors declare that the research was conducted in the absence of any commercial or financial relationships that could be construed as a potential conflict of interest.
